# Supplementary material for: Safely and autonomously cutting meat with a collaborative robot arm
Source: Sci Rep. 2024 Jan 2;14:299. doi: 10.1038/s41598-023-50569-4 (PMC10762048; doi:10.1038/s41598-023-50569-4)
Supplement: Supplementary file 1 — Supplementary Legends. [file 41598_2023_50569_MOESM1_ESM.pdf]

Legend for Supplementary Video

1. "Safely and autonomously cutting meat with collaborative robot arms"
   by Sagar Parekh, Ryan Wright, Robin White, and Dylan P. Losey
2. Current automation in the meat industry is highly specialized and inflexible, with robots separated from human co-workers.
3. We envision multi-purpose robot arms that collaborate with humans throughout meat processing.
4. First, we create a safety framework to ensure the robot arm is safe for nearby humans.
   We constrain the robot to move within a specified region above the cutting board…
   …and we develop and test an instrumented knife that can detect unexpected contacts.
5. Next, we create a vision and control approach to detect meat, plan autonomous robot cuts, and execute cutting trajectories.
   Our framework detects and localizes meat using a camera, then plans autonomous robot motions to process the meat.
6. We show that robots with this framework can slice strips of meat, remove excess fat, and make uniform cubes.
   Slicing: First the robot autonomously detects and slices a pork loin into 8 – 10 uniform strips.
   Point-to-point: A collaborative human uses markers to indicate where the robot should cut to remove fat.
   Trimming: Instead of relying on the human, the robot can also autonomously detect and remove fat.
   Cubing: Finally, the robot cuts the trimmed meat into cubes of equal size.
7. We measured the resulting meat products to assess the accuracy of our approach.
   Accuracy: The error between the robot's desired cuts and actual cuts was less than the industry standard.
   Slicing: The average weight of each slice was within the USDA suggested range.
   Removing Fat: An expert survey of the cuts suggested that point-to-point achieves better performance than trimming.
   Cubing: The average dimensions of the cubes lie within allowable error margins.
